# Calcium and Boron Fertilization Improves Soybean Photosynthetic Efficiency and Grain Yield

**DOI:** 10.3390/plants11212937

**Published:** 2022-11-01

**Authors:** Tatiani Mayara Galeriani, Gabriel Oliveira Neves, João Henrique Santos Ferreira, Rafael Neres Oliveira, Sirlene Lopes Oliveira, Juliano Carlos Calonego, Carlos Alexandre Costa Crusciol

**Affiliations:** Department of Crop Science, College of Agricultural Sciences, São Paulo State University (UNESP), Botucatu 18610-034, SP, Brazil

**Keywords:** soybean, foliar fertilization, flowering

## Abstract

Foliar fertilization with calcium (Ca) and boron (B) at flowering can promote flower retention and pod fixation, thereby increasing the number of pods per plant and, in turn, crop productivity. The objective of this work was to investigate the effects of Ca + B fertilization during flowering on the nutritional, metabolic and yield performance of soybean (*Glycine max* L.) The treatments consisted of the presence and the absence of Ca + B fertilization in two growing seasons. Crop nutritional status, gas exchange parameters, photosynthetic enzyme activity (Rubisco), total soluble sugar content, total leaf protein concentration, agronomic parameters, and grain yield were evaluated. Foliar Ca + B fertilization increased water use efficiency and carboxylation efficiency, and the improvement in photosynthesis led to higher leaf sugar and protein concentrations. The improvement in metabolic activity promoted a greater number of pods and grains plant^−1^, culminating in higher yields. These results indicate that foliar fertilization with Ca + B can efficiently improve carbon metabolism, resulting in better yields in soybean.

## 1. Introduction

Foliar fertilization is an alternative for nutritional management, mainly used as a nutritional supplement [1]. In soybean, foliar nutrient management was common during the reproductive period, when plants have high nutrient requirements due to the high transfer rates of nutrients and sugars for the formation of reproductive structures and, ultimately, grain production [2,3,4,5].

Foliar application of calcium (Ca) and boron (B) is widely used in Brazil [6,7], as they are essential nutrients for the growth and development of plants, in addition to regulating various physiological processes. The application of Ca and B, isolated or combined, has been exhaustively studied by several authors [6,8,9,10,11,12]; however, there are few studies under field conditions evaluating the combined effect of Ca + B on photosynthetic aspects in soybean crops.

Calcium is a structural component of cell walls and membranes, an intracellular messenger and necessary for the formation of new cells [13,14]. Ca is also an important signaler of auxins, which in turn reduce the abscission process of leaves, flowers, and fruits [15,16]. Boron is a structural element of cell walls and membranes [17] and functions in sugar transport and metabolism, lignification, meristem tissue cell division, flower and seed formation, and protein synthesis [18]. Several studies have shown that the combined application of Ca + B promoted greater vegetative development, increased grain and fruit quality and production in different crops compared to the isolated application of these elements [19,20,21,22,23]. These results suggest that these nutrients are co-limiting—when nutrients are provided in combination, they tend to have a greater response than when provided isolated, where, Ca + B > Ca or Ca + B > B as explained by Sadras et al. [24].

The supply of combined Ca and B in soybean flowering is an increasingly common practice among producers, due to its benefits in fruiting, as they are essential for the germination of pollen grains and pollen tube elongation, favoring fertilization and a reduction in flower abortion [6,9]. The associated supplementation of foliar Ca and B has promoted an increase in the number of pods per plant, resulting in higher productivity [7,8,11,25,26,27]. These effects are mediated by increased physiological and photosynthetic efficiency in terms of carbon assimilation and carbohydrate synthesis [28,29]. Thus, Ca and B are important not only in the reproductive phase but also throughout plant life, as they affect photosynthetic capacity, photoassimilate transport [29,30,31] and water absorption [17].

Weaver et al. [32] verified that applications of calcium nitrate and boric acid in bean flowering, provides better pod fixation and a high increase in grain yield. Bevilaqua et al. [33] and Souza et al. [34] also verified the increase in soybean grain yield with a combined application of Ca and B.

In this study, we proposed to evaluate whether the management performed by Brazilian farmers is effective in the fruiting process. We hypothesized that the combined application of Ca + B can improve photosynthetic metabolism and, consequently, increase fruit set, culminating in an increase in grain yield. Therefore, our study aimed to investigate the combined application of Ca + B that can improve the photosynthetic, nutritional, and productive metabolism of soybean.

## 2. Results

### 2.1. Climate Characteristics

**Figure 1:**
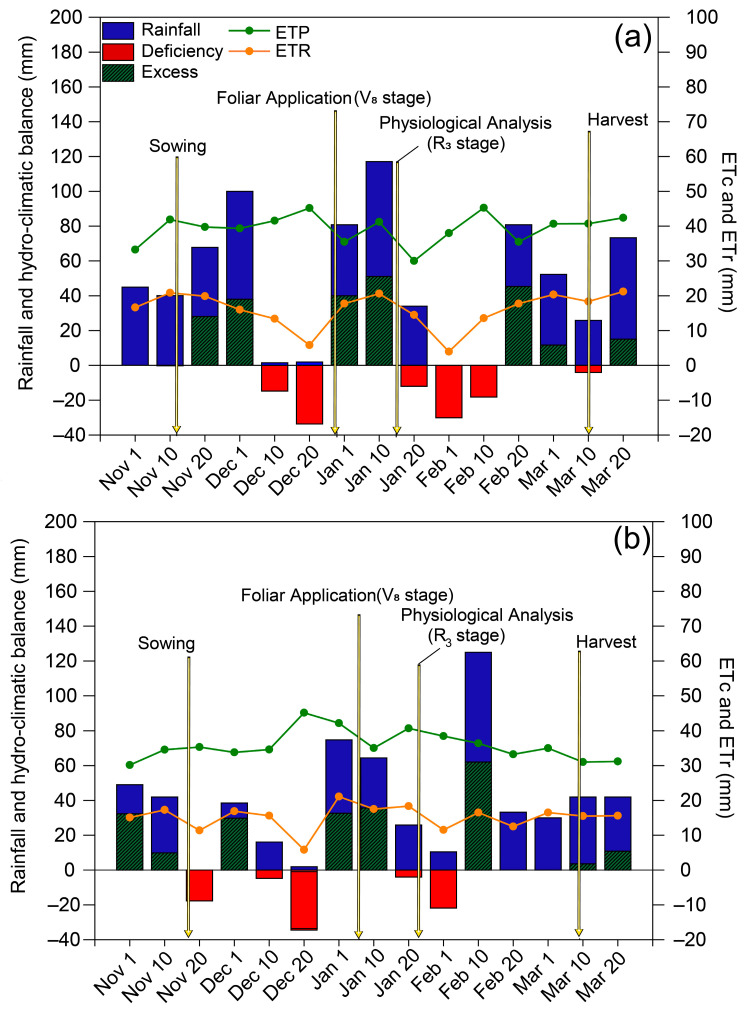
Climatological water balance at Botucatu (SP), Brazil, during the (**a**) 2018/2019 and (**b**) 2019/2020 soybean growing seasons. ETc: crop evapotranspiration; ETr: real evapotranspiration. The arrows indicate the timing of spraying and evaluations.

Rainfall in the 2018/2019 and 2019/2020 growing seasons was 569 and 434 mm, respectively (Figure 1). During the 2018/2019 growing season, there were two periods of low rainfall during the development of the soybean crop: the first occurred between the end of the vegetative stage and the beginning of flowering, while the second occurred between the end of flowering and the beginning of pod formation. Drought stress was less intense in the 2019/2020 season than in the previous season and occurred during the vegetative stage and between the end of phenological stage R_3_ and the end of phenological stage R_5_.

### 2.2. Nutritional Status, Gas Exchange and Carbon Metabolism

**Figure 2:**
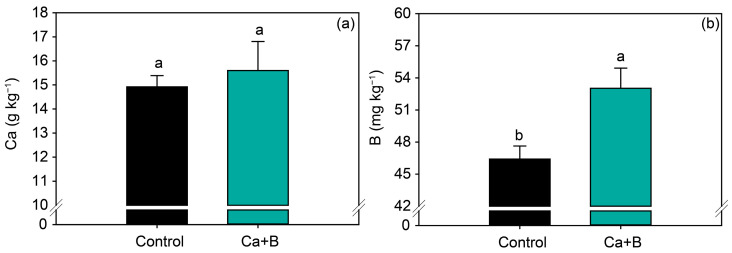
Soybean leaf levels of calcium (Ca) (**a**) and boron (B) (**b**) as a function of foliar application of Ca + B. Different lowercase letters indicate a significant difference between Ca + B treatments by Fisher’s test (*p* ≤ 0.05). Error bars express the standard error of the mean (*n* = 12).

**Figure 3:**
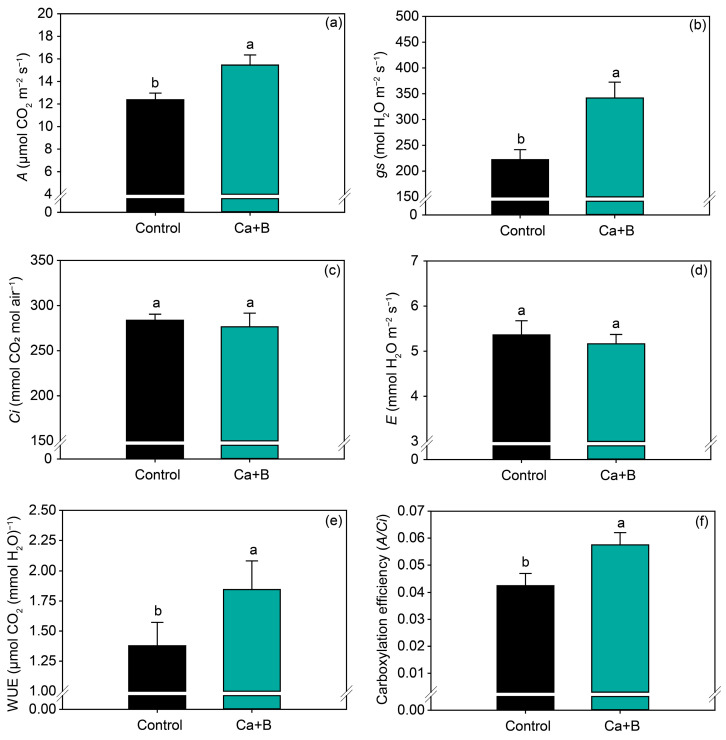
Net photosynthetic rate (*A*) (**a**), stomatal conductance (*gs*) (**b**), substomatal concentration of CO_2_ (*Ci*) (**c**), leaf transpiration (*E)* (**d**), water use efficiency (*WUE*) (**e**), and carboxylation efficiency (*A*/*Ci*) (**f**) as a function of foliar application of calcium (Ca) plus boron (B) in the 2019/2020 growing season. Different lowercase letters indicate a significant difference between Ca + B treatments by Fisher’s test (*p* ≤ 0.05). Error bars express the standard error of the mean (*n* = 12).

Foliar application of Ca + B did not affect the nutrients (N, P, K, Ca, Mg, S, Cu and Zn) (Figure 2a and Figure S1) but increased the leaf concentration of B by 14% (Figure 2b). Foliar application of Ca + B improved photosynthetic activity compared with the control. The net photosynthetic rate (*A*) and stomatal conductance (*gs*) increased by 25% and 18%, respectively (Figure 3a,b). As a result of the increase in *A*, water use efficiency (WUE) and carboxylation efficiency (*A*/*Ci*) increased by 34% and 35%, respectively, compared with untreated plants (Figure 3e,f). The internal concentration of CO_2_ (*Ci*) decreased by 2% (Figure 3c) in plants receiving foliar Ca + B application, further contributing to the improvement in *A/Ci*.

**Figure 4:**
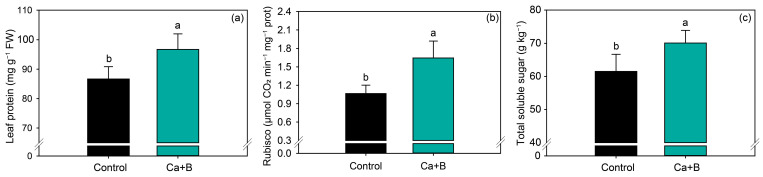
Leaf protein content (**a**), Rubisco activity (**b**) and total soluble sugar (**c**) as a function of foliar application of calcium (Ca) plus boron (B). Different lowercase letters indicate a significant difference between treatments Ca + B by Fisher’s test (*p* ≤ 0.05). Error bars express the standard error of the mean (*n* = 12).

The application of Ca + B increased leaf protein content by 12% (Figure 4a) and Rubisco activity by 54.4% compared with untreated plants (Figure 4b). Rubisco activity is closely associated with photosynthetic parameters, and thus the concentration of total soluble sugar in soybean leaves also increased by 14% compared with the control (Figure 4c).

### 2.3. Yield Components and Grain Yield

**Figure 5:**
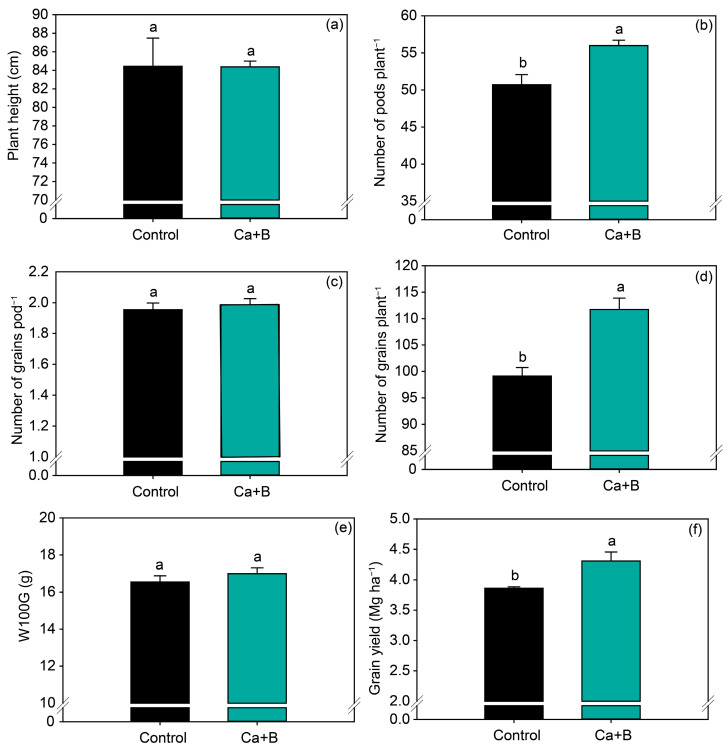
Production components: plant height (**a**), number of pods per plant (**b**), number of grains per pod (**c**), number of grains per plant (**d**), weight of 100 grains (W100G) (**e**) and grain yield (**f**) as a function of foliar application of calcium (Ca) plus boron (B). Different lowercase letters indicate a significant difference between Ca + B treatments by Fisher’s test (*p* ≤ 0.05). Error bars express the standard error of the mean (*n* = 12).

In general, the application of Ca + B increased soybean yield components. Fertilization with Ca + B increased the number of pods per plant and number of grains per plant by 10% and 13%, respectively (Figure 5b,d), but did not affect plant height, number of grains per pod, or W100G (Figure 5a,c,e). Consistent with the increase in the number of pods per plant, Ca + B application increased grain yield by 0.4 Mg ha^−1^ (Figure 5f) compared with untreated plants.

## 3. Discussion

Calcium and Boron play several important roles in plant metabolism [35] with structural and reproductive functions. Even when soil Ca and B levels are adequate for crop development, combined foliar application of these nutrients can enhance photosynthesis and increase the setting of flowers and pods, thereby increasing productivity. However, the underlying processes are not fully understood [36].

In the present study, foliar fertilization with Ca + B did not change the concentrations of leaf macronutrients but effectively increased the leaf B concentration. This increase was the result of rapid absorption of the applied B by soybean leaves, as cuticular membranes are highly permeable to uncharged, undissociated boric acid (H_3_BO_3_) [37,38,39]. Thus, foliar application of B can improve growth parameters by supplying this element to regions of growth, thus minimizing the effects of the low rate of redistribution of B in the plant. The range of B considered adequate for the development of dicotyledons is 20–70 mg kg^−1^ dry weight [40,41], and for soybean, the range of adequate B supply is 21–55 mg kg^−1^ [36]. According to these guidelines, the leaf concentrations of B observed in the present study are within the range of sufficiency for soybean. However, the ranges used for the interpretation of leaf analysis may not reflect the actual nutritional necessity of the crop, as they are old and require updating.

By contrast, foliar application of Ca + B did not change the soybean leaf concentration of Ca. Nutrient absorption studies show that soybean accumulates approximately 50 kg ha^−1^ at the beginning of the reproductive stage (R_1_) and an average of 120 kg ha^−1^ throughout its entire cycle [42,43]. Therefore, the application of small doses, as in the case of this study, 400 g ha^−1^, would hardly change the foliar contents of soybean as stated by Moreira et al. [44]. Therefore, the great benefit of applying Ca in small doses would be its stimulating effect, as some studies report positive effects on photosynthesis and productivity through the application of foliar Ca, although foliar Ca levels were not significantly altered [45,46,47,48]. The lack of change in the leaf Ca concentration may also be related to the low ability of the plant to redistribute this nutrient [10,49]. However, the values obtained were within the range of sufficiency for soybean (4.0–20 g kg^−1^) [36].

In response to foliar fertilization with Ca + B, an increase in photosynthetic activity was observed, induced by an improvement in gas exchange parameters and an increase in Rubisco activity. In this work, the improvement in photosynthetic activity is related to an increase in net photosynthesis and stomatal conductance and a decrease in the internal concentration of CO_2_ in the substomatic chamber (*Ci*). The net photosynthesis (*A*) is the result of the balance between simultaneous processes in which CO_2_ is fixed (carboxylation) and released (photorespiration, diurnal respiration) [50]. The *gs* indicates the stomatal opening and closing capacity, which consequently influences the flow of water and gases between the plant and the atmosphere, while *E* is characterized by the loss of water from the plant to the atmosphere as a function of the stomatal opening to diffusion of CO_2_ for photosynthesis. A reduction in *Ci* indicates that the CO_2_ that diffused into the substomatal chamber was assimilated into the mesophyll cells. Thus, higher values of *A* and *gs*, combined with low values of *Ci* and *E*, indicate greater efficiency in the assimilation of carbon and its consequent conversion into carbohydrates. Furthermore, the increase *A* promoted WUE, which is determined by the *A/E* ratio. This indicates that the plant was able to assimilate more carbon while consuming the same amount of water, since *E* was not changed [51].

In addition to gas exchange, the increase in *A* may have been influenced by the increase in rubisco activity, which may have been affected by the increase in the concentration of proteins in the leaves [38], considering that approximately 50% of the total soluble protein content in the leaves is composed of Rubisco [52].

Although the leaf concentration of Ca did not increase in treated plants, foliar fertilization with Ca likely stimulated improvements in plant photosynthetic activity, sugar translocation, and crop productivity [45,46,47,48,53,54] because Ca functions in photosynthetic pathways as a stomatal regulator controlling gas exchange [55,56]. There is evidence that B indirectly affects photosynthetic capacity, since several studies suggest that B can regulate the levels of chlorophyll, soluble proteins in leaves, photosynthetic enzymes, stomatal frequency and opening, the structure of chloroplasts and thylakoids and the electron transport chain (ETC) [37,57,58,59,60,61]. In addition, by acting on the transport of phloem of the plant, it improves the draining capacity and decreases the accumulation of sugar in the leaves, thus, B can act in the positive regulation of photosynthesis [17,62,63,64].

The improvement in photosynthesis may have been responsible for the observed increase in sugar concentration. This increase in the sugar concentration in the period before floral differentiation and pod formation directly reduces the abortion of reproductive structures and grain filling [65,66]. Thus, foliar application of Ca + B can stimulate plant physiological processes such as photosynthesis and increase grain production. The plants in the treatments that received foliar Ca + B had more pods and consequently more grains per plant [25,26,27]. Interestingly, Ca and B act in several processes that modulate the production and translocation of carbohydrates in plants [67,68]. The increase in total leaf sugar content before grain filling was efficiently redistributed to the developing organs, contributing to greater numbers of pods and grains per plant and higher yield.

In this study, the levels of Ca and B in the soil and in the plant [36] presented adequate levels for the soybean crop, but the supplementation with Ca + B improved the carbon metabolism, culminating in increases in productivity. This observation corroborates the concept of stimulating fertilization, because soybean productivity was increased even when these plants were already well nourished. These results support the management performed by Brazilian farmers, in which the supply of small doses of nutrients via foliar at strategic phenological stages can substantially contribute to the increase in yield in the high productivity of field crops.

## 4. Materials and Methods

### 4.1. Field Description

The experiment was conducted during the 2018/2019 and 2019/2020 soybean growing seasons at the Experimental Lageado farm of São Paulo State University (UNESP) in the southeastern region of São Paulo State, Brazil (22°51′ south, 48°26′ west, and 786 m above sea level). The soil in the experimental area is classified as a Latosol, clay-textured, kaolinitic, thermic typic Haplorthox [69]. The climate of the region is classified as Cwa (hot mesothermic temperate) [70], with rain in summer and drought in winter. The mean precipitation is 1.360 mm year^−1^, and the average annual temperature is 20.7 °C (mean of 50 years) [71]. Maximum and minimum temperature, precipitation and evapotranspiration data were collected from a meteorological station near the experimental area. The climatological balance was calculated according to the method proposed by Rolim [72].

Before establishing the experiment, the granulometric and chemical properties of the soil were determined at a depth of 0–20 cm (Table S1). Based on the soil chemical analysis, dolomitic limestone was used to increase the base saturation (V%) to 70%. The dolomitic limestone contained 280 g kg^−1^ calcium oxide (CaO), 200 g kg^−1^ magnesium oxide (MgO) and 81% calcium carbonate equivalent (CaCO_3_) as determined using the methodology of Quaggio and van Raij [73].

### 4.2. Experimental Design and Treatment Establishment

A randomized block design (RBD) was used with twelve blocks. The treatments consisted of foliar application or not of Ca + B in two seasons. The plots were composed of 10 rows with a length of 10 m each and an inter-row spacing of 0.45 m, resulting in an area of 45 m^2^. In both growing seasons, Ca + B application was performed at soybean phenological stage R_1_ (beginning of flowering) [74] as 400 g ha^−1^ Ca (Calcium chloride, CaCl_2_.2H_2_O) and 40 g ha^−1^ B (boric acid, H_3_BO_3_) plus organosilicon adjuvant at a dose of 30 mL ha^−1^ (polydimethylsiloxane, d = 1.1 g cm^−3^) (Ubyfol, Uberaba, Brazil). Applications were performed using a CO_2_ pressure box sprayer equipped with a 3.0 m boom and six flat fan nozzles (TTI 1102 VP, TeeJet, United States) regulated at an operating pressure of 1.8 bar, resulting in a spray volume of 150 L ha^−1^. The treatments were applied during the morning at temperatures of 25–29 °C, relative humidity of 75–80%, moist soil and wind speed of 6.0–8.0 km h^−1^.

### 4.3. Soybean Cultivation

Mechanized sowing was performed on 11 November 2018, and 17 November 2019. In both growing seasons, the soybean cultivar was TMG 7062 IPRO (Tropical Breeding & Genetics^®^). Seeds were treated with the fungicides carboxin (1 g i. a. kg^−1^ of seed) and thiram (1 g i. a. kg^−1^ of seed) (UPL, Campinas, Brazil) and inoculated with Semia 5079 (*Bradyrhizobium japonicum*) and Semia 5080 (*Bradyrhizobium diazoefficiens*) before sowing. Sowing was performed at a density of 14 seeds m^−1^, corresponding to a population of approximately 310,000 plants ha^−1^. Sowing fertilization was 60 kg ha^−1^ P_2_O_5_ and 60 kg ha^−1^ K_2_O in both growing seasons. Crop management during the experimental period followed the recommendations based on soybean phenological stage proposed by Cosmo et al. [75].

### 4.4. Assessment of Soybean Chemical and Physiological Parameters

#### 4.4.1. Crop Nutritional Status 

To determine the nutritional status of the soybean plants, the third fully expanded leaf with petiole from the apex to the base was collected from 20 plants per plot according to Ambrosano et al. [76] at phenological stage R_3_ (beginning of pod formation) [74]. The concentration of nitrogen (N) in the plant material was determined by sulfur digestion and the Kjeldahl distillation method. The leaf concentrations of potassium (K), Ca, magnesium (Mg), B, copper (Cu) and zinc (Zn) were determined by atomic absorption spectrometry after extraction by nitroperchloric digestion, whereas the leaf concentrations of phosphorus (P) and sulfur (S) were determined by colorimetry using the methodology proposed by Malavolta et al. [77].

#### 4.4.2. Gas Exchange Parameters

Gas exchange measurements were performed only in the 2019/2020 growing season using a model CIRAS-3 portable gas exchange device (PP Systems Inc., Amesbury, MA, USA). The readings started after the stabilization of the equipment with the temperature of the lead chamber adjusted to 28 °C, 380 ppm of CO_2_ and 1.000 µmol m^2^ s^−1^ of photosynthetically active radiation (PAR) provided by LED lamps. All readings were obtained between 8:00 and 10:00 a.m. The following parameters were measured in phenological stage R_3_: net photosynthetic rate (*A*, µmol CO_2_ m^−2^ s^−1^), stomatal conductance (*gs*, mol H_2_O m^−2^ s^−1^), internal CO_2_ (*Ci*, µmol mol^−1^), transpiration (*E*, mmol mol H_2_O m^−2^ s^−1^), water use efficiency (WUE, µmol CO_2_ (mmol H_2_O)^−1^) determined from *A*/*E*, and carboxylation efficiency determined from *A*/*Ci*.

#### 4.4.3. Total Concentration of Soluble Sugar

The total concentration of soluble sugar was measured at phenological stage R_3_ in both growing seasons using the phenol-sulfur method proposed by Dubois et al. [78], in which sugars are dehydrated in concentrated acid and subsequently complexed with phenol. In brief, 20 µL of supernatant was added to 0.5 mL of 5% phenol and 2 mL of sulfuric acid. The total concentration of soluble sugar was determined by reference to a standard sucrose curve and expressed in g kg^−1^.

#### 4.4.4. Total Leaf Protein Concentration

The total leaf protein concentration was determined at phenological stage R_3_ in both growing seasons. Proteins were extracted from 1.5 g of frozen plant material ground with a mortar and pestle under liquid nitrogen and suspended in 20% PVPP and extraction solution (100 mM potassium phosphate pH 7.5, 1 mM EDTA and 1 mM DTT). The homogenized material was centrifuged at 10,000 rpm for 25 min at 4 °C, and the supernatant was stored in Eppendorf tubes at −80 °C. The soluble protein concentration was determined bovine serum albumin (BSA) as a standard according to the method proposed by Bradford [79]. Aliquots of 100 µL of protein extract were mixed with 5 mL of Bradford reagent and analyzed in a spectrophotometer at 595 nm. Total protein content was determined by reference to a standard curve constructed using BSA and expressed as mg g^−1^ of fresh weight (FW).

#### 4.4.5. Photosynthetic Enzyme Activity

Ribulose-1,5-bisphosphate carboxylase/oxygenase enzyme activity (Rubisco) was measured in the third fully expanded leaf without petiole collected at the R_3_ stage in the 2019/2020 growing season only. The methodology described by Reid et al. [80] was used. Frozen plant material (3 g) was ground with a mortar under liquid nitrogen and suspended in 1.5 mL of extraction buffer (58 mM potassium phosphate and 1 mM EDTA). The homogenized material was centrifuged at 14,000 rpm for 25 min at 4 °C, and the supernatant was stored at 4 °C. The Rubisco incubation buffer consisted of 100 mM bicine-NaOH pH 8.0, 25 mM KHCO_3_, 20 mM MgCl_2_, 3.5 mM ATP, 5 mM phosphocreatine, 0.25 mM NADH, 80 kat glyceraldehyde-3-phosphate dehydrogenase, 80 kat 3-phosphoglyceric phosphokinase and 80 kat creatine phosphokinase. A 70-µL aliquot of supernatant was incubated with 900 µL of incubation buffer at 30 °C for 5 min in the absence of ribulose-1,5-bisphosphate (RuBP) to allow Rubisco carboxylation. The oxidation of NADP was initiated by adding 30 µL of 16.66 mM RuBP to the cuvette. The absorbance at 340 nm was measured in a spectrophotometer. Enzyme activity was determined by the difference between absorbance readings obtained at 0 and 1 min (without moving the instrument cuvette) and expressed in µmol min^−1^ mg protein.

#### 4.4.6. Agronomic Parameters and Grain Yield

After the plants reached physiological maturity, a useful area of 4.05 m^2^ was manually harvested in each plot (3 rows with a length of 3 m each) to evaluate plant height (cm), number of pods per plant, number of grains per pod, number of grains per plant, weight of 100 grains (W100G), and grain yield (Mg ha^−1^). Plant height was measured with a tape measure from the base of the plant near the ground to the top. The number of pods per plant and number of grains per pod were measured from the average of 10 plants, while the number of grains per plant was determined by dividing the number of grains per pod by the number of pods per plant. W100G was determined by weighing 100 grains, and grain yield was determined by weighing all grains harvested from the crop area and extrapolating to Mg ha^−1^. W100G and grain yield were corrected to 13% moisture on a dry basis. Moisture was determined using an automatic mini GAC meter [81].

### 4.5. Statistical Analysis

The ordered data obtained in the evaluations were subjected to the Shapiro–Wilk normality test [82] and the homoscedasticity test [83] (*p* ≤ 0.05), [84], followed by analysis of variance (ANOVA) by the F test (*p* ≤ 0.05) using the statistical package SAS [85]. Foliar application and growing season were not significant at *p* ≤ 0.05 for any of the variables (S2). Therefore, data were combined across growing seasons. Results are reported as means.

## 5. Conclusions

Foliar application of Ca + B increased the efficiency of C assimilation and sugar production in soybean leaves, resulting in increased pod production and grain yield. These effects also reflect the importance of Ca and B during the reproductive phase of soybean. The present study confirms that foliar fertilization with Ca + B at the beginning of soybean flowering is a viable practice for increasing C metabolism and ensuring pod formation and fixation, which directly increase soybean grain yield. Future research should focus on the isolated and combined effects of the two elements (Ca and B) on the plant, given their importance in the reproductive phase.

## Data Availability

The datasets analyzed during the current study are available from the corresponding author upon reasonable request.

## Supplementary Materials

The following supporting information can be downloaded at: https://www.mdpi.com/article/10.3390/plants11212937/s1. Figure S1: Foliar contents of N (a), P (b), K (c), Mg (d), S (e), Cu (f) and Zn (g) in soybean as a function of foliar application of calcium (Ca) plus boron (B); Table S1: Physicochemical attributes (0.0–0.2 m depth) before sowing; Table S2: Nitrogen (E), phosphorus (P), potassium (K), calcium (With), magnesium (Mg), sulfur (S), boron (B), copper (Cu), zinc (Zn), leaf protein, total soluble sugar (TS), plant height (PH), number of pods per plant (NPP), number of pods per grain (NGPod), number of grains per plant (NGP), 100 grains weight (W100G) and grain yield (Gy) as affected by cropping cycles and Ca + B foliar application.
