# Supplementary material for: Calcium and Boron Fertilization Improves Soybean Photosynthetic Efficiency and Grain Yield
Source: Plants (Basel). 2022 Nov 1;11(21):2937. doi: 10.3390/plants11212937 (PMC9657382; doi:10.3390/plants11212937)
Supplement: Supplementary file 1 [file plants-11-02937-s001.zip › plants-1947973-supplementary.pdf]

Article

Calcium and Boron Fertilization Improves Soybean Photosynthetic Efficiency and Grain Yield

SUPPLEMENTARY MATERIAL

Tables

Table S1. Physicochemical attributes (0.0 -0.2 m depth) before sowing.

Sand	Clay	Silt	Soil texture	Soil density
--------------- ($g\ kg^{-1}$) ---------------				(gcm^{-3})
117.0	602.0	281.0	Clay	1.19

pH	MO	resina P	K^{+}	Ca^{2+}	Mg^{2+}	SB	CTC	SB
($CaCl_2$)	($g\ kg^{-1}$)	($mg\ kg^{-1}$)	---------------------- ($mmol_c\ kg^{-1}$) --------------------------					%
5.4	26.2	29.0	3.9	25.0	15.0	43.9	86.0	51.0

S	Al^{3+}	H+Al	Fe	Cu	Mn	Zn	B
($g\ kg^{1}$)	($mmol_c\ kg^{1}$)		----------------------------- ($mg\ kg^{-1}$) ------------- ------------				
4.9	2.0	42.0	22.0	8.8	26.2	2.0	0.4

Table S2. Nitrogen (N), Phosphorus (P), Potassium (K), Calcium (Ca), Magnesium (Mg), Sulfur (S), Boron (B), Copper (Cu), Zinc (Zn), Leaf Protein, Total Soluble sugar (TS), plant height (PH), number of pods per plant (NPP), number of pods per grain (NGPod), number of grains per plant (NGP), 100 grains weight (W100G) and grain yield (GY) as affected by cropping cycles and Ca + B foliar application.

Treatments	N	P	K	Ca<br>g kg^{-1}	Mg	S	B	Cu	Zn
2019/2020	40.3 b	3.1 a	16,9 b	16,0 a	3,3 b	2,1 a	48,5 b	12,1 a	46,9 a
2020/2021	42.0 a	3.0 a	22,4 a	14,6 b	3,5 a	2,1 a	50,9 a	10,3 b	42,5 b
F probability									
Treatment (T)	0.252	0.350	0.428	0.611	0.215	0.017	<0,001	0.185	0.070
Years (Y)	0.003	0.210	<0,001	<0,001	0.002	0.468	0.004	0.006	<0,001
T × Y	0.739	0.455	0.009	0.299	0.817	0.141	0.624	0.249	0.755

Treatments	Protein	TS	PH	NPP<br>mg kg^{-1}	NGPod	NGP	W100G	GY
2019/2020	90.0 a	72.4 a	71.1 b	48.6 b	1.9 b	92.3 b	15.3 b	3.3 b
2020/2021	93.4 a	59.2 b	97.7 a	58.1 a	2.0 a	118.6 a	18.2 a	4.8 a
F probability								
Treatment (T)	0.000	<0.001	0.938	<0.001	0.073	<0.001	0.003	<0.001
Years (Y)	0.152	<0,001	<0,001	<0,001	<0,001	<0,001	<0,001	<0,001
T × Y	0.814	0.054	0.910	0.892	0.478	0.360	0.129	0.348

* Means followed by the same letters in the columns do not differ significantly by Fisher's test (p≤0,05).

Figure

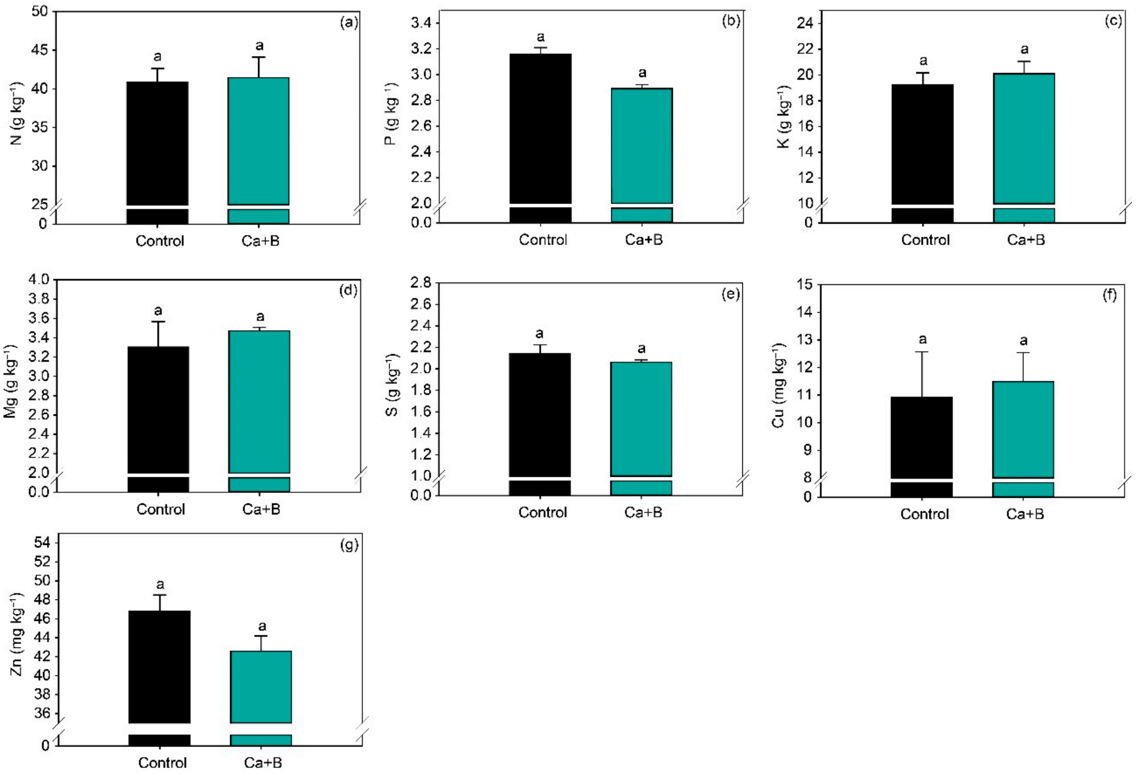

Figure S1. Foliar contents of N (a), P (b), K (c), Mg (d), S (e), Cu (f) and Zn (g) in soybean as function of foliar application of Calcium (Ca) plus Boron (B). Different lowercase letters indicate a significant difference between treatments (presence or absence of Ca + B) by Fisher's test ($p \leq 0.05$).
